# Separation of Chloride and Sulfate Ions from Desulfurization Wastewater Using Monovalent Anions Selective Electrodialysis

**DOI:** 10.3390/membranes14040073

**Published:** 2024-03-25

**Authors:** Xufeng Tian, Dongbei Yue, Tao Hou, Fuyuan Xiao, Zhiping Wang, Weibin Cai

**Affiliations:** 1 School of Environment, Tsinghua University, Beijing 100084, China; txf21@mails.tsinghua.edu.cn (X.T.); yuedb@tsinghua.edu.cn (D.Y.); 2 Horizon (Beijing) Environmental Engineering Co., Ltd., Beijing 101299, China; houtao@horwater.com; 3 School of Chemical and Environmental Engineering, China University of Mining and Technology, Beijing 100083, China; sqt2200303116@student.cumtb.edu.cn (F.X.); sqt2200303115@student.cumtb.edu.cn (Z.W.)

**Keywords:** electrodialysis, selective separation, desulfurization wastewater, ion-exchange membranes, permselectivity, energy consumption

## Abstract

The high concentration of chloride ions in desulphurization wastewater is the primary limiting factor for its reusability. Monovalent anion selective electrodialysis (S-ED) enables the selective removal of chloride ions, thereby facilitating the reuse of desulfurization wastewater. In this study, different concentrations of NaCl and Na_2_SO_4_ were used to simulate different softened desulfurization wastewater. The effects of current density and NaCl and Na_2_SO_4_ concentration on ion flux, permselectivity ($P_{{\mathrm{SO}}_{4}^{2-}}^{{\mathrm{Cl}}^{-}}$) and specific energy consumption were studied. The results show that Selemion ASA membrane exhibits excellent permselectivity for Cl^−^ and SO_4_^2−^, with a significantly lower flux observed for SO_4_^2−^ compared to Cl^−^. Current density exerts a significant influence on ion flux; as the current density increases, the flux of SO_4_^2−^ also increases but at a lower rate than that of Cl^−^, resulting in an increase in permselectivity. When the current density reaches 25 mA/cm^2^, the permselectivity reaches a maximum of 50.4. The increase in NaCl concentration leads to a decrease in the SO_4_^2−^ flux; however, the permselectivity is reduced due to the elevated Cl^−^/SO_4_^2−^ ratio. The SO_4_^2−^ flux increases with the increase in Na_2_SO_4_ concentration, while the permselectivity increases with the decrease in Cl^−^/SO_4_^2−^ ratio.

## 1. Introduction

The limestone-gypsum wet flue gas desulfurization technology is widely employed in thermal power plants [1,2,3,4,5], due to its high maturity, exceptional adaptability to diverse flue gas components, and stable operation [6,7]. During the desulfurization process, besides SO_2_, a certain amount of Cl^−^, F^−^, and heavy metal ions are also absorbed by the desulfurization slurry in the absorption tower. As the desulfurization solution circulates, these ions continuously accumulate and concentrate within the lime slurry. However, an increase in the Cl^−^ concentration can lead to various issues including reduced quality of desulfurization gypsum. When the Cl^−^ concentration reaches a certain level, it significantly diminishes desulfurization efficiency. In addition, it can also cause corrosion of pipelines and equipment. To ensure the desulfurization efficiency, controlling Cl^−^ concentration in the slurry is generally necessary. Different factories have varying maximum limits for Cl^−^ concentration; however, most adhere to levels below 20,000 mg/L [5,6].

The technologies for desulfurization wastewater treatment primarily encompass chemical precipitation [8,9,10], evaporative crystallization [11], spray evaporation [12,13], membrane separation [14,15,16], and biological treatment technology [17]. Chemical precipitation is the most commonly employed technology, which can remove suspended solids and heavy metal ions from wastewater efficiently. However, the treated wastewater still retains a high salt content, posing challenges for reuse. If discharged into the environment, it can have adverse effects [18]. Evaporative crystallization entails substantial investment and operating costs. The process of spray evaporation is relatively straightforward but encounters difficulties in maintaining operational stability and achieving powder treatment. Membrane separations, such as HERO and NF, are commonly employed for wastewater concentration to minimize the energy consumption of subsequent evaporation. However, these methods necessitate rigorous pretreatment procedures, including lime–soda softening or sodium hydroxide–soda softening, to completely eliminate calcium ions and prevent calcium sulfate scaling during membrane concentration [15,19].

Developing novel advanced technologies for desulfurization wastewater treatment holds significance in cost reduction while accomplishing cascade utilization of water resources in thermal power plants. As an efficient membrane separation technology, electrodialysis (ED) has been successfully applied across various fields [20,21,22,23,24,25,26]. When employing ED for desulfurization wastewater treatment, to prevent calcium sulfate scale formation and membrane fouling during concentration processes, it is necessary to soften the wastewater in advance [27,28,29]. However, due to the lack of selectivity during the concentration process, the obtained concentrated solution contains both high concentrations of NaCl and Na_2_SO_4_, requiring significant investments with intricate operations for subsequent fractional crystallization equipment.

The emergence of monovalent-ion-selective ion exchange membranes (IEMs) provides additional options for selective separation of different ions [30,31,32,33,34]. The monovalent selective IEMs exhibit considerable selectivity towards ions with different valence and size, thereby facilitating the separation and purification of diverse salts in brine. Reig et al. concentrated SWRO brine using S-ED with Neosepta cation and anion exchange membranes, resulting in a concentration increase from approximately 70 to 245 g/L NaCl while achieving intrinsic purification of major multivalent ions [35]. Sharma et al. enhanced the monovalent selectivity by fabricating a polyamide selective layer on commercial IEMs through interfacial polymerization (IP), which was then utilized for concentrating NaCl in seawater reverse osmosis brine. The results demonstrated that the IP-modified IEMs exhibited a divalent rejection rate exceeding 90%, whereas the commercial IEMs displayed a divalent rejection rate below 65% [36].

Yang et al. investigated separation performance of seawater reverse osmosis brine using S-ED and observed a decrease in specific energy consumption as temperature increased. However, there was a significant decline in the selectivity for monovalent and divalent ions [37]. Luo et al. proposed a two stage S-ED process to selectively separate F^−^ from ammonia-based flue gas desulfurization slurry, achieving an 81.4% total recovery rate for F^−^. The NH_4_F content increased from 0.35% to 40.70% [38].

In the case of limestone–gypsum wet flue gas desulfurization wastewater, the main restriction on the reuse of desulfurization wastewater is the Cl^−^ in the wastewater. Selective removal of chloride salts through S-ED enables recycling of the wastewater back into the desulfurization system. The concentrated water obtained through S-ED primarily consists of chlorine salt; however, if softened prior to undergoing S-ED treatment, sodium chloride becomes its primary component which simplifies further treatment.

The composition of desulfurization wastewater is complex and varies significantly among different thermal power plants in terms of salt content, Cl^−^, SO_4_^2−^, and other components’ concentration levels. The concentration of Cl^−^ is generally not less than 5000 mg/L and not more than 20,000 mg/L, but with some manufacturers, it can be as high as 40,000 mg/L [18]. The concentration of SO_4_^2−^ fluctuates more, and the higher concentration is close to 30,000 mg/L [5,8,10,28,39,40]. Most of the existing studies are focused on specific wastewater, such as seawater, seawater reverse osmosis concentrated water, and brackish water, and the concentration of SO_4_^2−^ is mostly lower than 6000 mg/L. The study on SO_4_^2−^ concentrations exceeding 6000 mg/L is limited. In addition, there is no systematic study on the separation characteristics of wastewater with different concentrations and proportions of Cl^−^ and SO_4_^2−^. To mitigate the impact of cations, varying concentrations of NaCl and Na_2_SO_4_ were employed to simulate softened desulfurization wastewater. For the treatment of desulfurization wastewater via S-ED, this study systematically examined how Cl^−^ and SO_4_^2−^ concentration as well as current density affect separation performance regarding Cl^−^ and SO_4_^2−^. The impact of Cl^−^ and SO_4_^2−^ concentration on limiting current density (LCD) was also studied.

## 2. Materials and Methods

### 2.1. Materials and Chemicals

The artificial brine was prepared using de-ionized water and chemicals including sodium chloride(NaCl) and anhydrous sodium sulfate(Na_2_SO_4_). The chemicals were all purchased from Aladding Regent (Shanghai) Co., Ltd., Shanghai, China, and all were analytical grade.

The anion exchange membrane (AEM) and cation exchange membrane (CEM) used in this experiment were Selemion ion exchange membranes manufactured by AGC Engineering Co., Ltd., Chiba, Japan, and were purchased from Shandong Tian Wei Membrane Technology Co., Ltd., Shandong, China. The specific membrane performance parameters are shown in Table 1.

### 2.2. Experimemtal Setup and Procedure

**Figure 1:**
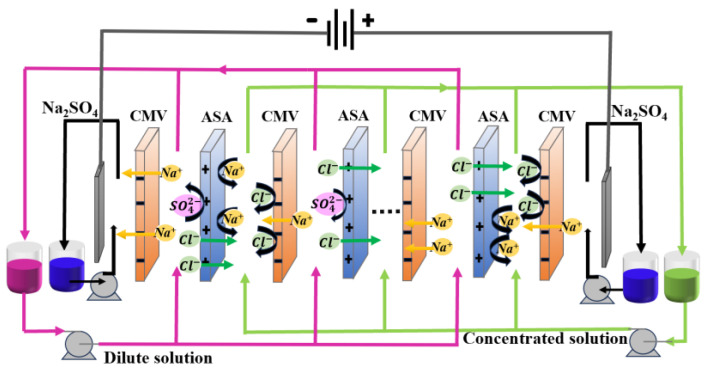
The schematic diagram of the monovalent anionic S-ED process and ions migration in membrane stack.

The self-assembled S-ED stack was employed in the experiments. Figure 1 presents the schematic diagram of the S-ED process and ions migration in the membrane stack. A DC power supply (WYK5020, Beijing Active Power Technology Co., Ltd., Beijing, China) was utilized to provide the separation force. Specifically, ruthenium-coated titanium served as the cathode, while stainless steel 316 L was used as the anode. The bench ED stack consisted of 5 cell pairs IEMs with each membrane having an effective surface area of 50 cm^2^ and dimensions of 10 cm × 5 cm. Diagonal net spacers (Beijing Sanyuan Bada Co., Ltd., Beijing, China) with a thickness of 0.85 mm were implemented to separate each pair of membranes, promoting fluid turbulence and reducing concentration polarization effects.

2 wt% sodium sulfite (Na_2_SO_4_) were used as the anode and cathode solution, respectively. The dilute compartment was filled with 500 mL NaCl + Na_2_SO_4_ solution, while the concentrate compartment was filled with 500 mL 0.3 wt% NaCl solution in order to decrease the electric resistance in the early stage of experiments. Both the desalting stream and concentrating stream were maintained at a flow rate of 40 L/h, corresponding to 5.2 cm/s. The electrolyte streams were also maintained at a flow rate of 40 L/h, corresponding to 5.7 cm/s. All experiment were conducted at room temperature and in a constant current mode. The current density was varied from 5 to 25 mA/cm^2^.

### 2.3. Analytical Methods

The concentrations of Cl^−^ and SO_4_^2−^ were determined using ion chromatography (ICS-1100; Thermo Dionex; column model: AS19 4 mm × 250 mm). The mobile phase was 20 mmol/L potassium hydroxide (KOH) solution, with a flow rate of 1.0 mL/min. The injection volume was 25 μL, and the column temperature was 30 °C.

### 2.4. Data Analysis

#### 2.4.1. Monovalent Selectivity Coefficient $P_{{\mathrm{SO}}_{4}^{2-}}^{{\mathrm{Cl}}^{-}}$

The permselectivity between Cl^−^ and SO_4_^2−^ was defined as Equation (1) [41]

(1) $$P_{{\mathrm{SO}}_{4}^{2-}}^{{\mathrm{Cl}}^{-}}=\frac{\left(c_{{\mathrm{Cl}}^{-},ct}V_{{\mathrm{Cl}}^{-},ct}-c_{{\mathrm{Cl}}^{-},c0}V_{{\mathrm{Cl}}^{-},c0}\right)/\left({c_{\mathrm{SO}}}_{4,ct}^{2-}{V_{\mathrm{SO}}}_{4,ct}^{2-}-{c_{\mathrm{SO}}}_{4,c0}^{2-}{V_{\mathrm{SO}}}_{4,c0}^{2-}\right)}{c_{{\mathrm{Cl}}^{-},d0}/{c_{\mathrm{SO}}}_{4,d0}^{2-}}$$

where *c_i_* is the concentration of the ions in the dilute compartment; *V_i_* is the volume of dilute or concentrate compartment; the subscripts *d* and *c* represent the dilute compartment and the concentrate compartment, respectively; *t* and 0 represent time *t* and 0, respectively.

#### 2.4.2. Removal Rate of Cl^−^

The removal rate of Cl^−^ (α) was calculated as Equation (2)

(2) $$\alpha =\frac{\left(c_{{\mathrm{Cl}}^{-},ct}V_{{\mathrm{Cl}}^{-},ct}-c_{{\mathrm{Cl}}^{-},c0}V_{{\mathrm{Cl}}^{-},c0}\right)}{c_{{\mathrm{Cl}}^{-},d0}V_{{\mathrm{Cl}}^{-},d0}}$$

#### 2.4.3. Ion Flux

The average ion flux (*J_i_*, mol/m^2^ h) was calculated as Equation (3) [42]

(3) $$J_{i}=\frac{\Delta c_{\mathrm{ct}}V_{\mathrm{ct}}}{AN\Delta t}$$

where *A* is the effective area of anion exchange membrane; *N* is the number of membrane pairs, here *N* equals 5; Δ*t* is the time interval is the time interval between two sampling points.

#### 2.4.4. Energy Consumption Per Unit of NaCl

The energy consumption in terms of product NaCl was calculated according to Equation (4) [41]

(4) $$E_{\mathrm{NaCl}}=\int \frac{UIdt}{C_{t}V_{t}M}$$

#### 2.4.5. Transport Number

Transport numbers *t_i_* were calculated according to Equation (5) [43]

(5) $$t_{i}=\frac{\left|z\right|J_{i}}{\sum_{i}^{n}\left|z\right|J}$$

where *z* represents the valence of the ion.

## 3. Results and Discussion

### 3.1. LCD for Different Concentration of NaCl and Na_2_SO_4_

Concentration polarization refers to the phenomenon of ion depletion that occurs at the interface between the membrane and solution during electrodialysis when the current density exceeds the maximum current density for stable mass transfer. The difference in diffusion rate of ions in both membrane and solution phases is an inherent factor leading to concentration polarization. LCD is of great significance for the operation of electrodialysis, as it theoretically requires electrodialysis to operate at an appropriate current density. Electrical resistance (Δ*V*/*i*) versus reciprocal of the current (1/*i*) was used to determine the LCD [37]. The testing method used was proposed by Cowan and Brown. The current was increased from 0 V manually until a turning point appears on the Δ*V*/*i*−1/*i* curve. 

**Figure 2:**
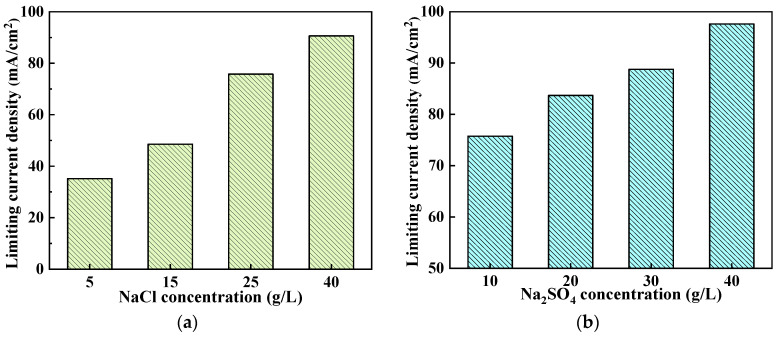
Limiting current density at different NaCl and Na_2_SO_4_ concentrations by the Cowan and Brown method: (**a**) 10 g/L Na_2_SO_4_ and different concentrations of NaCl, (**b**) 25 g/LNaCl and different concentrations of Na_2_SO_4_.

The concentration of chloride ions and sulfate ions in different desulfurization wastewater varies greatly. Therefore, the effect of different concentrations of NaCl and Na_2_SO_4_ on the LCD was studied. Firstly, by controlling the concentration of Na_2_SO_4_ at 10 g/L, the effect of NaCl concentration on the LCD was studied. The results are shown in Figure 2a. It can be seen that the concentration of Cl^−^ has a significant impact on the LCD. When the NaCl concentration is 5 g/L (equivalent to 3.03 g/L Cl^−^), the LCD is 32 mA/cm^2^. As the NaCl concentration increases, the LCD rises rapidly. When the NaCl concentration reaches 40 g/L (equivalent to 24.3 g/L Cl^−^), the LCD increases to 90.6 mA/cm^2^. Since the SO_4_^2−^ concentration remains basically unchanged during monovalent anionic S-ED process, Figure 2a can also represent LCD of saline water at different desalination stages. Although initial LCD is high, as desalination progresses and Cl^−^ concentration decreases, there is a rapid decline in LCD; therefore, attention should be paid to controlling current density in later stages of desalination process. The positive relationship between LCD and NaCl concentrations in the dilute compartment is consistent with Lee et al. [44]. Figure 2b shows the effect of SO_4_^2−^ concentration on the LCD when the concentration of NaCl is controlled to 25 g/L. Increasing Na_2_SO_4_ concentration leads to a slight rise in LCD, but this increase is relatively small due to greater resistance against SO_4_^2−^. For monovalent anion S-ED, the diffusion resistance of Cl^−^ is low, while that of SO_4_^2−^ is high. However, as the current density increases, the stack voltage drop and the driving force for SO_4_^2−^ also increase, thereby enhancing the diffusion rate of SO_4_^2−^. Near the LCD, where the stack voltage drop approaches approximately 20 V, a substantial portion of the current is attributed to sulfate ion diffusion. In this case, higher concentrations of SO_4_^2−^ result in faster sulfate diffusion rates and consequently lead to an increase in LCD. Furthermore, Figure 2b illustrates that as the concentration of SO_4_^2−^ increases, there is a downward shift in the curve. This can be attributed to an elevation in Na_2_SO_4_ concentration which enhances brine’s conductivity, resulting in a lower resistance and higher current.

### 3.2. Effect of Current Density

**Figure 3:**
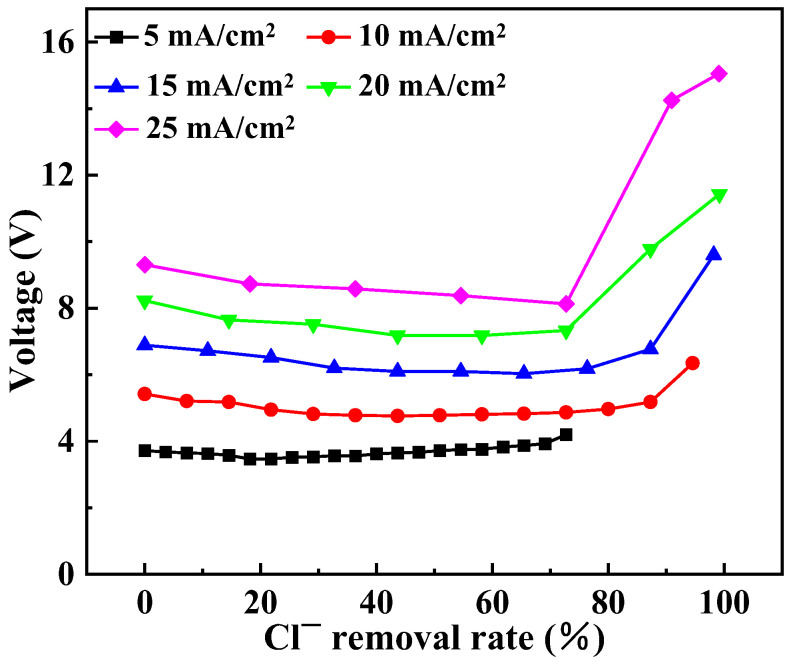
Variation in stack voltage drop with Cl^−^ removal rate at different current densities.

In the process of electrodialysis, current density plays a crucial role and is typically controlled within 90% of the maximum current density. Higher current density results in fast desalination speed and requires a smaller membrane area, but it also leads to higher energy consumption. Conversely, lower current density leads to slower desalination speed, larger required membrane area, but lower energy consumption. In addition, for monovalent anionic S-ED, it is necessary to study the effect of current density on the separation performance of Cl^−^ and SO_4_^2−^. Therefore, a systematic study on the impact of current density is needed. The experiment was conducted using a simulated solution containing 25 g/L NaCl and 20 g/L Na_2_SO_4_, and the current density is controlled within 80% of the maximum current. Figure 3 shows how stack voltage drop varies with Cl^−^ removal rate under different current densities. It can be observed that the higher current densities result in higher voltage. This can be attributed to the fact that voltage is directly proportional to current when the resistance remains constant. At the beginning of electrodialysis, as Cl^−^ removal rate increases, there is a gradual decrease in stack voltage drop; however, as the removal rate continues to increase further changes in stack voltage drop become relatively small; once Cl^−^ removal rate reaches approximately 75–90%, membrane stack voltage accelerates with increasing Cl^−^ removal rate. The reason for this is that a high Cl^−^ removal rate leads to a significant decrease in the LCD. When the current density exceeds the LCD, the stack voltage drop increases rapidly. The rapid increase in stack voltage drop leads to an accelerated SO_4_^2−^ flux which subsequently decreases permselectivity. Henceforth in subsequent experiments, Cl^−^ removal rate was controlled to 70%, and the corresponding NaCl concentration in the dilute compartment was about 7.5 g/L. Compared with Figure 2, it can be seen that all the corresponding LCD are greater than 35 mA/cm^2^, and the maximum current density in the following experiment was only 25 mA/cm^2^, which was within the appropriate current density range.

**Figure 4:**
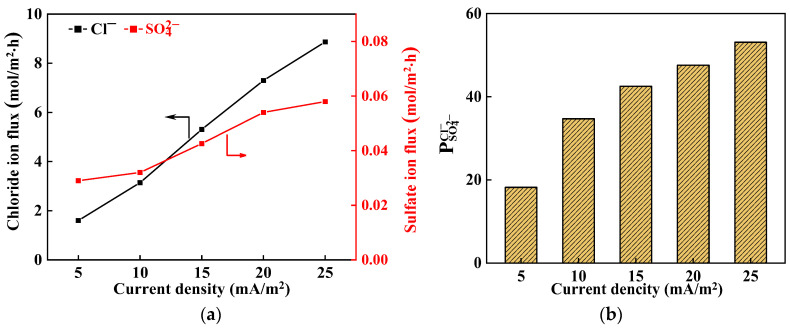
Influence of current density on (**a**) average ion flux and (**b**) permselectivity.

Figure 4a shows the impact of current density on average ion flux. It can be observed that the flux of Cl^−^ increases nearly linearly as the current density rises from 5 to 25 mA/cm^2^. According to Nernst-Planck equation, the ion flux can be defined as follows:

(6) $$J_{I}=-D_{i}\left(\nabla c_{i}+\frac{z_{i}F}{RT}c_{i}\nabla \varphi \right)$$

where *D_i_* is the diffusion coefficient of ions *i*; *c_i_* is the concentration; *c_i_* is the valence of ion *i*, F is the Fraday constant; and *φ* is the electrical potential. According to Equation (6), the ion flux is primarily influenced by concentration gradient, potential difference, and ion properties [45]. As the current density increases, so does electric potential difference (stack voltage drop), resulting in an increase in ion flux.

The average flux of SO_4_^2−^ is significantly lower than that of Cl^−^ within the experimental range. Specifically, the molar flux of SO_4_^2−^ accounted for only 0.6–1.8% compared to Cl^−^. This disparity can be primarily attributed to Donnan exclusion, dielectric effect, and steric effect. Within IEMs, there are three main factors that control ion flux, including Donnan exclusion, dielectric effect, and steric effect. Due to Donnan exclusion from the surface modification layer on the IEMs, the permeation resistance for SO_4_^2−^ is large. Additionally, the hydrated SO_4_^2−^ exhibits a larger size compared to the hydrated Cl^−^, resulting in enhanced steric effect and dielectric effect that impose greater resistance during passage through the IEMs. The combination of these three factors greatly increases the permeation resistance of SO_4_^2−^. 

Furthermore, as current density increases, the SO_4_^2−^ flux exhibits an increasing trend. This is primarily because the electric field also increases with current density leading to an enhanced driving force for sulfate ions under its influence. When this driving force partially compensates for the electrostatic repulsion of the modified layer, the growth of SO_4_^2−^ flux is accelerated.

Figure 4b shows the influence of current density on permselectivity. It can be observed that an increase in current density positively impacts the permselectivity. Specifically, as the current density rises to 25 mA/cm^2^, the permselectivity reaches its maximum value of 50.4. It should be pointed out that it is not true that the higher the current density, the higher the permselectivity, it holds true only within a specific range. Golubenko et al. [46] studied the influence of current density on permselectivity of monovalention of surface-sulfonated anion exchange membranes. Permselectivity ($P_{{\mathrm{SO}}_{4}^{2-}}^{{\mathrm{Cl}}^{-}}$) coefficients increase along with current density, reaching maximum values at the LCD. A simplified mass transfer and numerical simulations in the framework of Nernst–Planck–Poisson equations was developed. The model agrees well with the experimental data and also shows that permselectivity reaches the maximum values at LCD.

Gorobchenko et al. [47] introduced activity coefficients and developed a more detailed model for monovalent permselectivity of bilayer ion exchange membrane. According to this model, the increase in the permeability of a bilayer membrane with increasing current density at low voltages is due to the fact that the divalent ion cannot pass the barrier of the monovalent-selective layer. As the current density increases in this voltage range, the monovalent ion flux increases faster than the divalent ion flux. However, when the flux of the monovalent ion approaches its LCD, the rate of the increase of its flux slows down. When this flux reaches its limiting (nearly maximum) value, the flux of the divalent ion starts to grow, and the permselectivity coefficient starts to decrease.

The effect of current density on the transport number of Cl^−^ and SO_4_^2−^ is illustrated in Table 2. It can be observed that as the current density increases, the transport number of Cl^−^ rises while the transport number of SO_4_^2−^ decreases. This phenomenon can be attributed to the accelerated growth of Cl^−^ flux with increasing current density, resulting in an increase in transport number. Within the experimental range, the transport numbers of Cl^−^ are all greater than 0.9651, indicating excellent selectivity for monovalent ions by the ASA ion exchange membrane.

**Figure 5:**
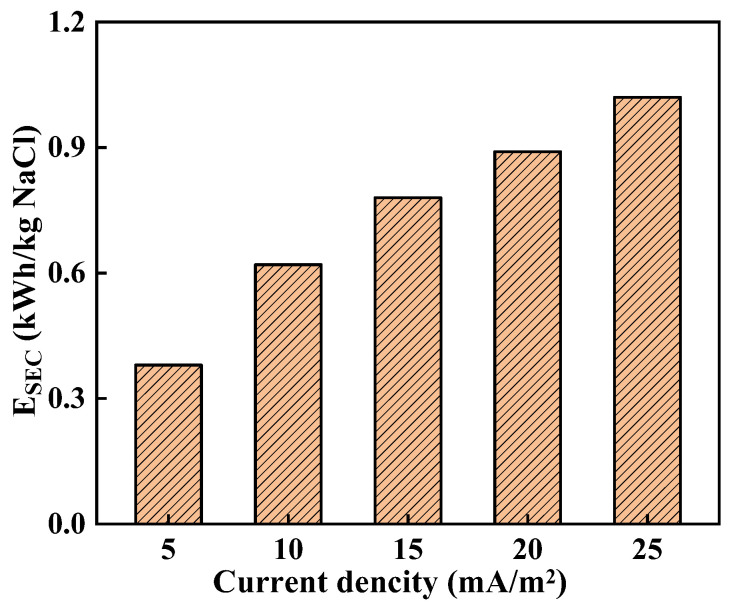
Influence of current density on specific energy consumption.

The effect of current density on *E_SEC_* when the Cl^−^ removal rate is 70% is illustrated in Figure 5. With an increase in current density, *E_SEC_* exhibits a near-linear growth pattern. This can be attributed to the approximate proportionality between *E_SEC_* and stack voltage, as indicated by Equation (4). Consequently, as current density rises, the voltage also experiences a linear increment, leading to a corresponding increase in *E_SEC_*. Notably, Yang et al. [37] and Zhang et al. [48] have reported similar findings regarding the significant impact of current density on *E_SEC_*. Overall, elevating the current density results in higher values for the stack voltage drop, permselectivity, and *E_SEC_*.

When the current density is low, despite the low energy consumption, the desalination time becomes excessively long, and permselectivity decreases. As the current density increases to 15 mA/cm^2^, an appropriate level of permselectivity is achieved while maintaining moderate energy consumption. Therefore, for subsequent experiments, a current density of 15 mA/cm^2^ was chosen.

### 3.3. Effect of NaCl Concentration

**Figure 6:**
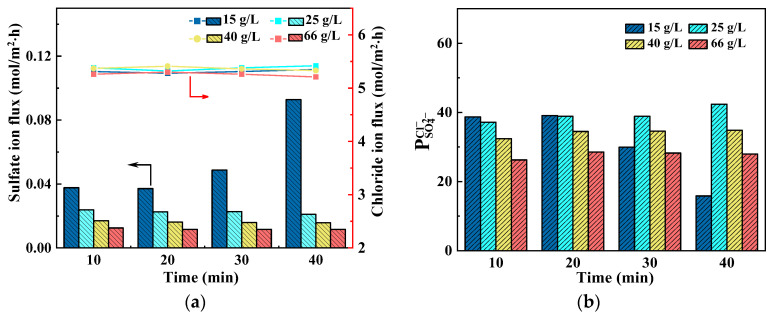
Effect of NaCl concentration on (**a**) average ion flux and (**b**) permselectivity at current density of 15 mA/cm^2^.

By maintaining the Na_2_SO_4_ concentration at 10 g/L and current density at 15 mA/cm^2^, the influence of NaCl concentration on SED was studied. Figure 6 shows the variation in ion flux and permselectivity over time. The Cl^−^ flux remains relatively unaffected by NaCl concentration, as shown in Figure 6a, while it exerts a significant impact on SO_4_^2−^ flux. With increasing concentration of NaCl, there is a gradual decrease in SO_4_^2−^ flux. The total flux of Cl^−^ and SO_4_^2−^ is determined by the current density and current efficiency. When the current density remains constant, and there is minimal variation in current efficiency, the overall flux experiences negligible changes. Since the flux of SO_4_^2−^ is significantly smaller than that of Cl^−^, the total flux is primarily determined by the latter. Therefore, when there is little change in the total flux, the flux of Cl^−^ remains relatively stable. On the other hand, as NaCl concentrations increase, solution resistance decreases along with a reduction in stack voltage drop which leads to a decrease in driving force on SO_4_^2−^. Henceforth, SO_4_^2−^ flux diminishes accordingly. 

Throughout the experimental duration, both Cl^−^ and SO_4_^2−^ fluxed remained basically unchanged, except when using a NaCl concentration of 15 g/L where an evident increase in SO_4_^2−^ flux was observed starting from the 30th minute onward. This is for the relatively low content of NaCl. After operating for 30 min, the remaining concentration of NaCl in the dilute compartment dropped below 7 g/L, leading to a noticeable increase in stack voltage drop and, consequently, an augmented SO_4_^2−^ flux. When operated for 40 min, with even less NaCl remaining and higher membrane stack voltage drop, the SO_4_^2−^ flux experiences further enhancement.

The effect of NaCl concentration on permselectivity is illustrated in Figure 6b. It is evident that an increase in NaCl concentration leads to a decrease in permselectivity. Despite the decrease in SO_4_^2−^ flux with increasing NaCl concentration, Equation (1) indicates that an increased denominator resulting from the increase in NaCl concentration causes a reduction in permselectivity.

The effect of NaCl concentration on the transport number of Cl^−^ and SO_4_^2−^ can be seen in Table 3 at the time of 20 min. The concentration of NaCl has obvious impact on the transport number, as evidenced by the experimental results. With increasing NaCl concentration, the transport number of SO_4_^2−^ shows a significant decrease, while the transport number of Cl^−^ keeps increasing. This is due to the significant decrease in SO_4_^2−^ flux with the increase in NaCl concentration.

### 3.4. Effect of Na_2_SO_4_ Concentration

**Figure 7:**
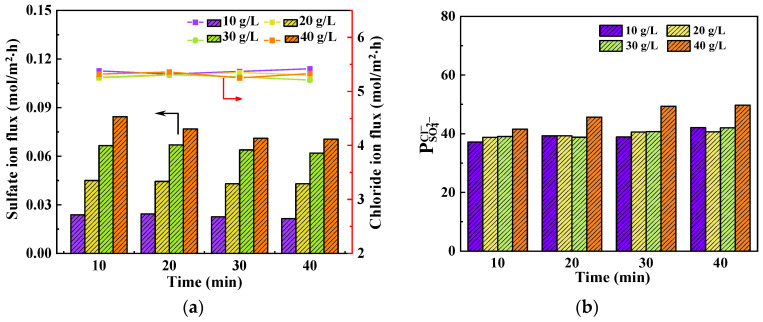
Effect of Na_2_SO_4_ concentration on (**a**) average ion flux and (**b**) permselectivity at current density of 15 mA/cm^2^.

The temporal variation in ion flux and permselectivity for different Na_2_SO_4_ concentrations is depicted in Figure 7. The concentration of NaCl was maintained at 25 g/L, while the current density was set to 15 mA/cm^2^. As shown in Figure 7a, the impact of Na_2_SO_4_ concentration on Cl^−^ flux is negligible, whereas it exerts a significant influence on SO_4_^2−^ flux. The effect on Cl^−^ flux is similar to that discussed in Section 3.3 and is not detailed here. For the SO_4_^2−^ flux, Equation (6) demonstrates that an increase in Na_2_SO_4_ concentrations leads to an amplified total driving force and subsequently enhances the SO_4_^2−^-flux. Throughout the experimental duration, both Cl^−^ and SO_4_^2−^ fluxes remained relatively stable, which is similar to Figure 6a.

Figure 7b shows the effect of Na_2_SO_4_ concentration on the permselectivity, revealing that permselectivity slightly increases with the increase of Na_2_SO_4_ concentration. Although the flux of SO_4_^2−^ increases with the increase in Na_2_SO_4_ concentration, according to Equation (1), the denominator also decreases. When the denominator decreases at a faster rate than the numerator, it will cause the increase in permselectivity.

The effect of current density on the transport number of Cl^−^ and SO_4_^2−^ can be seen in Table 4 at time of 20 min. The transport number is greatly influenced by the concentration of Na_2_SO_4_. As the Na_2_SO_4_ concentration increases, there is a significant increase in the transport number of SO_4_^2−^, while the transport number of Cl^−^ decreases continuously. This is due to the obvious increase in SO_4_^2−^ flux with the increase in Na_2_SO_4_ concentration.

## 4. Conclusions

In this study, monovalent anion S-ED was utilized for the separation of NaCl from the simulated desulphurization wastewater. Selemion ASA and CMV were selected as anion and cation ion exchange membrane, respectively. The effects of current density, NaCl concentration, and Na_2_SO_4_ concentration on the separation performance were systematically investigated. Results demonstrated that the ASA anion exchange membrane exhibited exceptional permselectivity and effectively removed chlorine salts. Within the experimental range, the transport number of SO_4_^2−^ is limited to a narrow range, ranging from 0.0044 to 0.0349, which facilitated subsequent treatment and utilization of NaCl. Increasing current density positively influenced separation performance. When increasing current density from 5 to 25 mA, permselectivity rose from 18.2 to 50.4; however, energy consumption also increased accordingly. Under fixed current density, the Cl^−^ flux remained relatively stable with increasing NaCl concentration, while both the SO_4_^2−^ flux and permselectivity decreased simultaneously. During the early stages of S-ED desalination, there was no significant change in Cl^−^ or SO_4_^2−^ flux or permselectivity over time. When NaCl concentration decreased to approximately 7 g/L, the SO_4_^2−^ flux increased rapidly, and permselectivity decreased accordingly. As Na_2_SO_4_ concentration increased, the SO_4_^2−^ flux also increased slightly along with a slight increase in selectivity coefficient. This study provides valuable insights for applying S-ED technology to desulfurize wastewater.

## Figures and Tables

**Table 1:** Main performance parameters of Selemion ion exchange membranes.

Characteristic		CMV	ASA
Thickness (μm)		120	120
Counter ion		Na^+^	Cl^−^
Burst strength (MPa)		0.16	0.14
Exchange capacity (meq g^−1^ dry)		2.01	2.0–2.1
Resistance (Ω cm^2^)	0.5 M NaCl	3	3.7
	0.5 M Na_2_SO_4_		13

**Table 2:** Influence of current density on transport number.

Current Density (mA/cm^2^)	5	10	15	20	25
*t* (Cl^−^)	0.9651	0.9800	0.9842	0.9854	0.9871
*t* (SO_4_^2−^)	0.0349	0.0200	0.0158	0.0146	0.0129

**Table 3:** Influence of NaCl concentration on transport number.

NaCl Concentration (g/L)	15	25	40	66
*t*(Cl^−^)	0.9861	0.9916	0.9941	0.9956
*t*(SO_4_^2−^)	0.0139	0.0084	0.0059	0.0044

**Table 4:** Influence of Na_2_SO_4_ concentration on transport number.

Na_2_SO_4_ Concentration (g/L)	10	20	30	40
*t*(Cl^−^)	0.9909	0.9835	0.9754	0.9721
*t*(SO_4_^2−^)	0.0091	0.0165	0.0246	0.0279

## Data Availability

The original contributions presented in the study are included in the article, further inquiries can be directed to the corresponding author.
